# Challenges in Managing Pregnancy in Patients With Fructose 1, 6‐Bisphosphatase Deficiency: A Single‐Centre Experience

**DOI:** 10.1002/jmd2.70116

**Published:** 2026-08-13

**Authors:** Raashda A. Sulaiman, Ibrahim Alqasir, Dana Alqasabi, Ruqaiah AlTassan, Maha Tulbah, Maha Alnemer, Mohamed Al‐Owain

**Affiliations:** ^1^ Department of Medical Genomics Genomic Medicine Center of Excellence, King Faisal Specialist Hospital and Research Center Riyadh Saudi Arabia; ^2^ College of Medicine Alfaisal University Riyadh Saudi Arabia; ^3^ Department of Nutrition King Faisal Specialist Hospital and Research Center Riyadh Saudi Arabia; ^4^ Department of Obstetrics and Gynecology King Faisal Specialist Hospital and Research Center Riyadh Saudi Arabia

**Keywords:** fructose 1,6‐bisphosphatase deficiency, gestational diabetes, hypoglycemia, lactic acidosis, pregnancy

## Abstract

Fructose‐1,6‐bisphosphatase deficiency is a rare inherited metabolic disorder of gluconeogenesis characterized by recurrent hypoglycemia and lactic acidosis, typically triggered by inadequate glucose intake or increased consumption of fructose, sucrose, or sorbitol. During pregnancy, the risk of hypoglycemia and lactic acidosis increases because endogenous glucose production is limited while fetal glucose demand rises. Here we report three patients with fructose‐1,6‐bisphosphatase deficiency who had successful pregnancies and deliveries. These patients were managed by a multidisciplinary team of obstetricians, maternal‐fetal medicine specialists, metabolic physicians, and metabolic dieticians. All patients were obese; one had morbid obesity. Two patients developed gestational diabetes mellitus. One responded to dietary intervention, whereas the patient with morbid obesity had poor dietary adherence, suboptimal glycemic control, and experienced an episode of severe life‐threatening hypoglycemia with lactic acidosis requiring intensive care. She recovered fully, with no adverse maternal or fetal effects. All patients had normal vaginal deliveries and were closely monitored during labor, delivery, and the postpartum period. Both patients with gestational diabetes developed lactic acidosis immediately after delivery despite stable blood glucose levels. Managing gestational or type 2 diabetes in patients with fructose‐1,6‐bisphosphatase deficiency is particularly challenging because the disorder predisposes them to hypoglycemia. This case series contributes to the limited literature on pregnancy outcomes and highlights key management challenges in this rare condition.

## Key Points

- Management of gestational diabetes in patients with fructose 1,6‐bisphosphatase deficiency requires a careful approach, maintaining a delicate balance between hyperglycemia and hypoglycemia, avoiding lactic acidosis.

## 1 Introduction

Fructose 1, 6‐bisphosphatase deficiency (FBPase; OMIM 229700) is a rare inherited autosomal recessive disorder, caused by a biallelic pathogenic variant in the *FBP1* gene. FBPase is the rate‐limiting key enzyme in the gluconeogenesis process, which produces endogenous glucose from non‐carbohydrate substrates, including lactate, glycerol, fructose, and glucogenic amino acids, to maintain normal blood glucose levels during fasting, starvation, and physical exertion. Patients with FBPase deficiency depend on glucose intake and hepatic glycogen degradation to maintain glucose homeostasis. They develop ketotic hypoglycemia and lactic acidosis following stress such as prolonged fasting, vomiting, diarrhea, fever, intercurrent infection, or high fructose, sorbitol, or sucrose intake. They usually remain asymptomatic between episodes.

During pregnancy, glucose metabolism is adapted to balance maternal nutrition with fetal development. There is increased peripheral insulin sensitivity in the first trimester, and fasting blood glucose levels tend to be on the lower side. However, when needed, this is compensated for by endogenous glucose production in a healthy woman [1, 2]. Later in the third trimester, insulin resistance increases, promoting endogenous glucose production and ensuring a steady glucose supply across the placenta for fetal growth [1]. Patients with FBPase deficiency with limited endogenous glucose production are at high risk of developing severe hypoglycemia and lactic acidosis during pregnancy when their oral intake is reduced due to nausea and vomiting.

To date, there are only four case reports on the management of pregnancies in patients with FBPase deficiency [3, 4, 5, 6]. The incidence of FBPase deficiency is relatively high in Saudi Arabia due to high consanguinity rates, and a cohort of 39 adult patients attends the metabolic clinic at our tertiary care facility. Here we report on three patients who underwent pregnancy and delivery at this centre. This case series describes successful management strategies for pregnant patients with FBPase deficiency, highlighting potential complications that may arise during pregnancy and the puerperium. These patients were diagnosed in early life, and their demographic data are shown in Table 1.

**TABLE 1:** Demographic features and course of pregnancies in three patients.

	Case 1	Case 2	Case 3
Age at diagnosis	4 years	1 month	8 months
Consanguinity	+	+	+
*FBP1* mutation (homozygous)	c.114_119dup; p.C39_T40 dup.	c.841G>T; p. Glu281*	c.841G>T; p. Glu281*
Co morbidities	Morbid obesity (BMI 40 kg/m^2^) Hepatic steatosis Prediabetes	Obesity (BMI 33 kg/m^2^) Hepatic steatosis Multinodular goiter Bilateral nephrolithiasis	Obesity (BMI 33.5 kg/m^2^) Hepatic steatosis Ulcerative colitis
Pregnancy course	GDM, episode of severe metabolic decompensation	Hypoglycemia in 1st trimester, GDM, COVID‐19 infection at delivery	Hypoglycemia in 1st trimester
Weight gain in pregnancy	13 kg	10.8 kg	6 kg
Fetal and maternal outcome	Satisfactory	Satisfactory	Satisfactory

Abbreviations: BMI, body mass index; GDM, gestational diabetes mellitus.

## 2 Case: 1

### 2.1 Preconception Period

This 33‐year‐old patient with morbid obesity (Table 1) followed an unrestricted diet and did not use uncooked cornstarch (UCCS) in adulthood. Despite recurrent hypoglycemia, she required hospitalization only twice in the preceding 10 years. Before pregnancy, she lost 12 kg with diet and exercise; early diet‐related hypoglycemia improved over time. Her husband's *FBP1* gene sequencing confirmed he was not a carrier of this disorder.

### 2.2 Pregnancy Course

The patient presented to the metabolic clinic at 9 weeks of gestation and was referred to a high‐risk pregnancy clinic, where an ultrasound confirmed a singleton pregnancy. She restricted fruit intake, took small meals rich in complex carbohydrates, and monitored blood glucose regularly, which showed fasting levels of 4.5–5.5 mmol/L, with hypoglycemic episodes (nadir 3.0 mmol/L) occurring only when meals were skipped. Serial ultrasounds at 18, 21, and 23 weeks showed normal anatomy, though estimated fetal weight (EFW) exceeded the 90th percentile. By 23 weeks, blood glucose levels were elevated (fasting 6.5–7.5 mmol/L; 2 h postprandial up to 13 mmol/L), consistent with gestational diabetes mellitus (GDM). She declined insulin and took metformin 500 mg three times daily with twice‐weekly lactate monitoring. After 1 week, lactate increased to 3.69 mmol/L (N < 2.0), prompting elective admission. The patient was asymptomatic with a blood glucose of 4.7 mmol/L; metformin was discontinued, 10% dextrose IV was administered, and lactate normalized. With dietary intervention, inpatient blood glucose ranged from 4.1 to 6.9 mmol/L. The patient was readmitted at 30 + 5 weeks for recurrent home hyperglycemia (8.6–11.9 mmol/L). During admission, her glucose levels stabilized (fasting: 5.2 mmol/L; 2‐h post‐meal: 6.7 mmol/L) with dietary intervention alone.

After discharge, the patient skipped meals to avoid hyperglycemia and returned the next day to the emergency department with nausea, vomiting, poor intake, lethargy, tachycardia, and tachypnea. Blood glucose was 2.7 mmol/L with severe high–anion‐gap metabolic acidosis and hyperlactatemia (venous blood gas: pH 7.11, pCO_2_ 2.6, bicarbonate 6.2 mmol/L, lactate 7.9 mmol/L); renal function and electrolytes were normal except for bicarbonate (7 mmol/L). The patient was admitted to the ICU and treated with 10% dextrose in half‐normal saline plus 40–60 mmol sodium bicarbonate/L at 120 ml/h, with close monitoring of glucose, lactate, and electrolytes. Hyperglycemia was managed with sliding‐scale insulin; prophylactic enoxaparin was given. Fetal monitoring remained reassuring, and dexamethasone was administered for fetal lung maturity. A multidisciplinary team decided to continue the pregnancy to allow further fetal maturation while prioritizing maternal stabilization and fetal surveillance. The patient recovered after three ICU days. Blood glucose was stable on insulin degludec 10 U daily, and she was discharged after 9 days with planned induction at 38 + 5 weeks.

### 2.3 Labor, Delivery and Post‐Natal Period

Labor was induced with prostaglandins and augmented with oxytocin. Blood glucose, lactate, and electrolytes were monitored closely. She received maintenance 10% dextrose in half‐normal saline with satisfactory blood glucose levels (4.5–10 mmol/L) and delivered a healthy male infant weighing 4020 g. Immediately after delivery, the patient developed metabolic acidosis (bicarbonate 15 mmol/L, lactate 6 mmol/L, glucose 7 mmol/L). Adding 40 mmol sodium bicarbonate to the dextrose infusion normalized lactate to 1.5 mmol/L within hours. The neonate was euglycemic.

The patient remained stable with no further lactic acidosis or hypoglycemia, was discharged on postpartum day 4, and continued to do well. Home blood glucose monitoring was satisfactory, and HbA1c was 5.5% (37 mmol/mol) 3 months after delivery.

## 3 Case: 2

### 3.1 Preconception Period

This 30‐year‐old patient with comorbidities (Table 1) was metabolically stable, except for recurrent hypoglycemia during a brief period when she was on weight‐reduction diet. She followed a partially fructose‐restricted diet without UCCS and was clinically and biochemically euthyroid, with stable renal and hepatic function. Her husband was a carrier of the same disorder. Her first pregnancy, managed abroad, was complicated by GDM with frequent hypo‐ and hyperglycemia and resulted in spontaneous vaginal delivery of an affected daughter. Her second pregnancy ended in a first‐trimester miscarriage.

### 3.2 Pregnancy Course

This patient presented to the metabolic clinic at 17 weeks gestation during her third pregnancy. There were several hypoglycemic episodes during the first trimester abroad. The patient was advised to eat regular meals, take nighttime UCCS (15 g), monitor fasting and 2‐h post‐meal blood glucose, and was referred to high‐risk obstetric care. Prenatal genetic testing was not performed because she presented at 17 weeks, and termination is not permitted in Saudi Arabia after 19 weeks' gestation [7].

The patient declined a 75‐g oral glucose tolerance test (OGTT) at 24 weeks' gestation. Serial fasting and 2‐h post‐meal blood glucose levels were normal (4.5–6 mmol/L), with HbA1c 5% (31 mmol/mol). She continued a low‐glycemic‐index diet, used nighttime UCCS, avoided prolonged fasting, and experienced occasional hypoglycemia (3–3.5 mmol/L) when meals were delayed. Fetal growth scans were normal until 34 weeks, when accelerated growth was noted (EFW ~2994 g, > 95th percentile). Fasting blood glucose remained normal (4.5–5 mmol/L), but 2‐h post‐meal levels rose to 9–11 mmol/L. She received further advice on complex carbohydrate meals. Given prior difficult‐to‐control GDM and excessive fetal growth, induction was planned at 38 weeks.

### 3.3 Labor, Delivery, Postnatal Period

The patient presented at 37 + 2 weeks in spontaneous labor with premature rupture of membranes and mild flu‐like symptoms; COVID‐19 PCR was positive. She remained metabolically and hemodynamically stable on supportive care. She received 10% dextrose with close monitoring of glucose, lactate, and renal profile, and had one vomiting episode without hypoglycemia. The patient delivered a healthy male infant vaginally, weighing 3210 g. Immediately after delivery, she developed metabolic acidosis (bicarbonate 16 mmol/L, lactate 3.6 mmol/L, glucose 8.2 mmol/L), which resolved after adding 40 mmol sodium bicarbonate per liter of 10% dextrose. The patient remained metabolically stable during the postnatal period, did not require oxygen or antiviral therapy for COVID‐19, and was discharged on postpartum day 3. The neonate was admitted to NICU for close blood glucose monitoring, and later genetic testing confirmed a heterozygous carrier status.

## 4 Case: 3

### 4.1 Preconception Period

This 25‐year‐old patient with comorbidities (Table 1) had been clinically stable for several years without hypoglycemia or metabolic acidosis. She followed a fructose‐restricted diet, did not use UCCS, and was metabolically stable. *FBP1* gene sequencing of her husband was unremarkable.

### 4.2 Pregnancy Course

The patient presented to the metabolic clinic at 8 weeks' gestation after experiencing several nocturnal hypoglycemic episodes while abroad. She was advised to monitor fasting and 2‐h post‐meal blood glucose, take regular CarboCh with UCCS, and was referred to high‐risk obstetric care. Ultrasound confirmed a singleton pregnancy. The patient took frequent small meals and preferred 1 cup of cooked corn 2–3 times daily instead of UCCS, which prevented hypoglycemia. CarboCh was later discontinued, and she remained stable without further hypoglycemia. OGTT at 24 weeks was normal; serial ultrasounds showed normal fetal growth and anatomy. Ulcerative colitis remained in remission on the same dose of mesalamine.

### 4.3 Labor, Delivery, Postnatal Period

The patient had spontaneous labor at 40 weeks and received 10% dextrose in saline with close monitoring of glucose, renal function, and lactate. She delivered a healthy female infant weighing 3420 g. The postpartum period was unremarkable; she resumed her pre‐pregnancy diet and was discharged in 3 days.

## 5 Discussion

This case series highlights the risks of hypoglycemia and lactic acidosis during pregnancy in women with fructose‐1,6‐bisphosphatase (FBPase) deficiency. To our knowledge, this is the first report describing pregnancy management in patients with FBPase deficiency complicated by GDM and severe metabolic decompensation.

These patients were diagnosed and followed at this tertiary care centre. The *FBP1* gene variants found in these patients (Table 1) were first reported by this centre in Saudi Arabia [8]. Patient 1 had a homozygous nonsense variant (c.841G>T), encoding a premature protein truncation (p. Glu281*) in the active site of the FBP1 enzyme. Patients 2 and 3 had a homozygous 6‐base‐pair duplication at positions 114 to 119, which encodes a duplication of two amino acids, cysteine and threonine (p.Cys39_Thr40dup), in the N‐terminal domain of FBP1 (Table 1). This is the first reported amino acid duplication in patients with FBPase deficiency. These *FBP1* variants are frequently found in Saudi patients with FBPAse deficiency and are associated with a severe phenotype.

Adult patients at our centre are advised to follow a diet rich in complex carbohydrates, restricted in fructose and sucrose. Frequent meals, 4–6 h daily, are advised. UCCS is recommended for patients with frequent hypoglycemia or during periods of reduced food intake and pregnancy; however, they do not always follow the instructions. The dose of UCCS is calculated for each individual case (average: 15–60 g/dose) and is to be given at night and as needed. Compliance with dietary instructions is monitored by their clinical and biochemical stability. They are advised to keep a food diary and regularly monitor blood glucose at home. Continuous glucose monitoring (CGM), if available, is an invaluable tool for detecting silent hypoglycemia. Regular blood tests for glucose, lactate, and renal function are performed at each clinic visit.

Despite their comorbidities (Table 1), these patients were metabolically stable before pregnancy. Hypoglycemic episodes occurred mainly when they reduced dietary intake in an attempt to lose weight. During routine follow‐up consultations, these women received preconception counseling to optimize pregnancy outcomes. The discussion addressed maternal and fetal risks, metabolic control, nutritional status, and the prevention of pregnancy‐related complications. It also included partner's genetic testing, particularly in consanguineous families, as well as reproductive options such as preimplantation genetic testing and prenatal diagnosis. *FBP1* gene sequencing was performed on the partners of all patients.

During pregnancy, these patients were followed regularly in the high‐risk pregnancy clinic by obstetricians and maternal‐fetal medicine specialists, in close collaboration with metabolic physicians and metabolic dieticians. Each patient had a personalized care plan for pregnancy, labor, delivery, and the postpartum period, documented in the electronic medical record to facilitate easy access by the multidisciplinary team (Table 2).

**TABLE 2:** Management plan for pregnant patients with fructose 1, 6‐bisphosphatase deficiency.

Antenatal period
Regular meals containing complex carbohydrates. Avoid prolonged fasting, high fructose, sorbitol, and sucrose intake.
Uncooked cornstarch supplements as needed. High‐glucose drinks are used when a routine diet cannot provide enough calories.
Regular home glucose monitoring to avoid hypoglycemia. Continuous glucose monitoring, if available, is preferred to capillary blood testing by glucometer.
If unwell, unable to take orally, visit the emergency room for blood testing, urinalysis for ketones and dextrose infusion as needed. Identify and treat metabolic stressors such as infection.
Labor
Monitor blood glucose, lactate and renal profile, including bicarbonate, at the onset of labor and 6 hourly thereafter.
Start a 10% dextrose infusion in half‐normal saline at the maintenance rate.
If oral intake is allowed, give high‐glucose drinks such as CarboCh.
Monitor blood glucose 4 hourly with a glucometer and maintain levels between 6 and 10 mmol/L. If blood glucose is consistently higher than 10 mmol/L, start low‐dose insulin by sliding scale to maintain it within the desired range.
Avoid ringer's lactate infusion.
If the patient develops metabolic acidosis and lactate rises, consult the metabolic team. Sodium bicarbonate may need to be added to the dextrose infusion.
Epidural analgesia is recommended to avoid the physiological stress of pain.
Postpartum period
Adequate pain management.
Close monitoring of blood glucose initially 4 hourly; electrolytes and lactate levels 6–8 hourly.
Close blood glucose monitoring of the newborn to detect hypoglycemia
Continue dextrose infusion till oral feeding is fully established.
Monitor for signs of hypoglycemia and metabolic acidosis (lethargy, vomiting, hyperpnoea)
Allow breastfeeding with a proportional increase in the mother's caloric intake.

Adult patients with FBPase deficiency in our cohort tend to have a high body mass index (BMI). Maternal obesity increases the risk of GDM. Hyperglycemia in GDM is driven by β‐cell dysfunction and severe peripheral insulin resistance. Increased placental transport of glucose, amino acids, and fatty acids stimulates fetal production of insulin and insulin‐like growth factor 1 (IGF‐1), contributing to fetal overgrowth [9, 10]. Other maternal and neonatal complications include preeclampsia, preterm delivery, neonatal hypoglycemia, and congenital deformities. A one‐step, two‐hour 75 g oral glucose tolerance test (OGTT) is performed in every pregnancy to screen for GDM between 24 and 28 weeks of gestation. Diagnosis is made when any glucose value meets or crosses the specified thresholds: fasting ≥ 5.1 mmol/L, 1‐h ≥ 10.0 mmol/L, or 2‐h ≥ 8.5 mmol/L [9, 10]. Two patients (1 and 2) developed GDM. Patient 1 had prediabetes (Table 1), and by 24 weeks, home monitoring of blood glucose showed high levels, confirming GDM. Patient 2 refused OGTT; had recurrent GDM characterized by 2 h postprandial hyperglycemia by the 34th week with acceleration in fetal weight.

Managing GDM or type 2 diabetes mellitus in patients with FBPase deficiency is challenging because the underlying primary disorder predisposes them to hypoglycemia. In these patients, continuous glucose monitoring (CGM) is preferred over point‐of‐care capillary testing because it provides a 24 h glucose profile that can detect unrecognized hypoglycemia or hyperglycemia that may be missed with intermittent finger‐prick testing; however, CGM was unavailable. If diet and exercise do not achieve adequate glycemic control, pharmacologic therapy is recommended. Insulin is the standard treatment for GDM uncontrolled by nonpharmacologic measures [9]; however, it may further increase hypoglycemia risk in these patients. Patient 2 responded to dietary intervention, whereas patient 1 had poor dietary adherence and suboptimal glycemic control. This patient declined insulin and chose metformin instead. Metformin is widely used as first‐line therapy for type 2 diabetes with obesity because it carries a low risk of hypoglycemia and weight gain. It effectively improves peripheral insulin sensitivity, increases peripheral glucose uptake, and suppresses hepatic gluconeogenesis. However, its precise mechanism of action is not fully understood. It reduces mitochondrial adenosine triphosphate (ATP) production, thereby increasing 5′adenosine monophosphate (AMP) levels and activating AMPK (AMP‐activated protein kinase), which inhibits FBPase activity in the liver and reduces hepatic gluconeogenesis [11]. Impaired gluconeogenesis also reduces lactate clearance [12]. Although these side effects are very rarely seen with a therapeutic dose of metformin, patients with FBPase deficiency may develop significant hypoglycemia and lactic acidosis due to further suppression of residual FBPase activity by metformin. Patient 1 received metformin with close lactate monitoring; it was discontinued after 1 week when lactate levels began to rise. Her case underscores the need for careful medication selection in FBPase deficiency, particularly avoiding agents that may precipitate lactic acidosis. During admissions, her glucose levels stabilized with dietary intervention alone. However, after discharge, she skipped meals to keep glucose within target ranges, resulting in severe hypoglycemia with lactic acidosis requiring ICU admission. The patient recovered fully, with no adverse maternal or fetal effects, and later had uncomplicated labor and delivery. Her son is now 2 years old with normal growth and development. Recurrent hypoglycemia and lactic acidosis may cause irreversible brain injury in both mother and fetus [1]. Both patients with GDM gained more weight than the recommended 5–9 kg for obese women [13].

Delivery planning and mode of delivery in these patients should be individualized based on maternal and fetal status, obstetric history, and any obstetric indication for cesarean section. All patients had normal vaginal delivery. During labor, delivery, and the immediate postpartum period, all patients received 10% dextrose in half‐normal saline at a maintenance rate, with close monitoring of blood glucose, electrolytes, and lactate levels. Despite maintaining normoglycemia and receiving epidural anesthesia to reduce physical exertion, patients 1 and 2 developed lactic acidosis immediately after delivery. This was managed by adding sodium bicarbonate to the dextrose infusion.

During vaginal delivery, physical exertion and uterine contractions may impair oxygenation and promote anaerobic glycolysis, leading to lactate accumulation in both mother and fetus [14, 15]. Patient 2 also had a mild COVID‐19 infection during labor that did not require specific treatment. All patients breastfed their babies and were advised to take an extra 300–400 kcal daily to support breastfeeding. They had no further complications during the remainder of the puerperium.

## 6 Conclusion

This case series adds to the literature on managing severe metabolic decompensation and GDM in women with FBPase deficiency, highlighting the challenges encountered by both the patients and the health care providers in pregnancy management. Comprehensive preconception counseling of patients on close glucose monitoring and preventing hypoglycemia is essential for better pregnancy outcomes. The development of GDM further complicates management as the primary disorder predisposes to hypoglycemia. Successful pregnancy outcomes depend on specific dietary intervention, avoidance of prolonged fasting, and close glucose monitoring, preferably by CGM. Clinicians must balance the risk of severe hypoglycemia against the maternal and fetal risks of GDM, including congenital anomalies, macrosomia, preterm birth, and pre‐eclampsia. Careful medication selection is important to prevent lactic acidosis in these patients. A coordinated multidisciplinary approach by a team of high‐risk obstetricians, maternal‐fetal medicine specialists, metabolic physicians, and metabolic dieticians, supported by a personalized electronic care plan covering pregnancy, labor, delivery, and postpartum care, is essential to optimize maternal and fetal outcomes.

## Author Contributions

**Raashda A. Sulaiman:** participated in the management of these patients, prepared their personalized care plan during pregnancy and labor, planned and wrote the manuscript. **Ibrahim Alqasir:** collected the data. **Dana Alqasabi:** participated in dietary management and contributed to the manuscript. **Ruqaiah AlTassan**, **Maha Alnemer**, **Maha Tulbah**, **Mohamed Al‐Owain:** participated in the management of patients and critically reviewed the manuscript.

## Funding

The authors have nothing to report.

## Ethics Statement

Research Advisory Council of the King Faisal Specialist Hospital and Research Center approved this research project (RAC # 2221255).

## Consent

Informed consent was obtained from all patients for inclusion in this study.

## Conflicts of Interest

The authors declare no conflicts of interest.

## Data Availability

Data are stored on a secure hospital server with no external access. Before sharing any data, we need the relevant permissions.
